# Impact of Replicated Biomimetic Microstructures on the Wettability of Injection-Molded Polymer Surfaces

**DOI:** 10.3390/biomimetics10110759

**Published:** 2025-11-11

**Authors:** Vojtěch Šorm, Jakub Bittner, Petr Lenfeld, Dora Kroisová, Štěpánka Dvořáčková

**Affiliations:** Faculty of Mechanical Engineering, Technical University of Liberec, 461 17 Liberec, Czech Republic

**Keywords:** biomimetics, relief, hierarchical microstructures, wettability, micro-milling, injection molding

## Abstract

This article evaluates the influence of replicated natural structures, produced by micro-machining, on the wettability of plastic parts made from hydrophilic and hydrophobic polymer materials under various temperature and pressure conditions. Although many studies have focused on biomimetic surface design, the effect of specific processing parameters on the accurate replication of natural topologies and their resulting wettability has been only partially explored. This study addresses this gap by systematically analyzing the effect of melt temperature and packing pressure on the functional replication of micro-machined biomimetic structures. The research describes the design of hierarchical microstructures inspired by biomimetics and their fabrication by micro-milling on molded parts. Test samples were prepared from polypropylene (PP), acrylonitrile butadiene styrene (ABS), and polyamide 6.6 (PA 6.6) under different processing parameters, and wettability was assessed using contact angle (CA) measurements. The results confirmed significant variations in surface wettability depending on polymer type, melt temperature, and packing pressure. For the hydrophilic relief (Rock Moss), contact angles below 90° were obtained for all tested polymers, including PP, which decreased from 98.7° on a flat surface to 82.4° at 220 °C and 500 bar. In PA 6.6, a reduction of up to 12% in contact angle was observed compared to smooth samples at 310 °C and 500 bar. For hydrophobic reliefs (Three-part Hibiscus and Tricolor Pansy), contact angles exceeded 100–110°, with the highest value of 108.3 ± 1.6° for PP at 200 °C and 500 bar. Suitable combinations of melt temperature and packing pressure enabled accurate replication of microstructures while preserving their functional wettability, demonstrating the possibility of tuning surface properties through topological design.

## 1. Introduction

Biomimetic surfaces represent one of the most dynamically developing areas of materials engineering. Their goal is to transfer the evolutionarily optimized functions of biological interfaces into technical applications and to use a combination of chemical composition and hierarchical topography (from nanoscale to microscale) to control wettability, adhesion, friction, heat transfer, and light transmission with high efficiency. A key benefit of this approach is that it achieves the desired functional properties without the need to apply permanent low-energy coatings, such as fluorinated polymers, which are environmentally problematic. An iconic example of the biomimetic approach is the so-called “lotus effect,” discovered by Barthlott and Neinhuis, where the micro- and nano-relief of the lotus leaf surface dramatically reduces the actual contact area between the drop and the substrate and promotes the Cassie-Baxter state, so that the drops roll off the dirt and the surface self-cleans [1,2]. This principle has become the basis for a number of patents and commercial products using self-cleaning or antifouling surfaces [2,3].

From a technological point of view, current production methods for biomimetic surfaces can be divided into “top-down” and “bottom-up” approaches. Top-down methods include laser texturing, such as laser-induced periodic surface structures (LIPSS/DLIP) [4,5]. These techniques enable the creation of submicrometer reliefs with optical and wetting functionalities on a wide range of materials used for the production of mold cavities in a single step [4,5,6]. Thanks to the possibility of applying a topological structure to the mold insert, they are compatible with mass production of polymer parts [6]. When combined with compression microinjection technology, low-cost replication of biomimetic surfaces is ensured [7]. These approaches enable the transfer of hierarchical topographies onto polymer surfaces with high precision and repeatability, allowing control over surface functions such as wettability, adhesion, or light transmission [8,9]. Recent studies have demonstrated that process parameters of injection molding, especially melt temperature, mold temperature, and packing pressure, play a decisive role in the replication fidelity and in the resulting surface functionality [8,9,10,11]. Bottom-up methods include chemical processes such as wet and gas phase etching, anodic oxidation, sol–gel processes, or self-organization of nanoparticles. In the last decade, SLIPS (slippery liquid-infused porous surfaces), inspired by the surface of the carnivorous plant Pitcher plant (*Sarracenia*), have also gained popularity [12,13]. These surfaces combine a nanoporous substrate with a lubricating liquid and exhibit universal, self-repairing, and transparent behavior with applications in biology and energy [14,15]. Masato et al. [16] provided a detailed review of surface texturing technologies for injection molding, including laser processing, lithographic methods, and mechanical machining.

The range of applications for biomimetic surfaces is extremely wide and includes, in particular: self-cleaning and antifouling surfaces—so-called lotus-inspired textures or “Sharklet” microstructures for suppressing the colonization of microorganisms [17]. Anti-icing and icephobic surfaces—superhydrophobic and SLIPS structures that reduce ice adhesion and facilitate de-icing [18]. Furthermore, antibacterial topography—mechanobactericidal nanoneedles inspired by the wings of dragonflies or cicadas [19]. Furthermore, “Optical anti-reflective moth-eye structures”—produced by nanoimprinting and used in optoelectronics or solar cells and microfluidic applications—control of wettability and capillary phenomena in lab-on-a-chip systems [20,21]. Recent studies have shown that controlled replication of hierarchical structures can significantly modify the wetting behavior of polymer parts and allow for the design of surfaces with tailored hydrophobic or hydrophilic properties [22,23]

The practical relevance of biomimetic surfaces in the polymer industry is particularly high [24]. The replication of micro- and nanostructures during injection molding depends on the geometry of the mold, the temperature of the melt and the mold, the size of the back pressure and the filling speed, and of course the type of polymer used [25]. These parameters directly influence the resulting wettability and functionality of the surface [6,7]. The experimental results obtained confirm that the optimization of injection conditions is key to achieving the desired surface behavior [6].

This research aims to experimentally and theoretically analyze the process of creating biomimetic surfaces with an emphasis on their replication in polymer materials. The study focuses on the structure topology design and preparation of microstructures on mold inserts, on the evaluation of the influence of injection molding parameters on the quality of structure transfer, and on the characterization of the resulting surfaces in terms of wettability. Although many studies have examined the wettability of microstructured polymer surfaces, most have focused on surface treatment or structural design rather than on the technological replication of natural topologies during injection molding. There is still a lack of comprehensive data showing how processing parameters such as melt temperature and packing pressure affect the replication fidelity and resulting wettability of various polymers. The present work addresses this gap by providing a detailed analysis of these effects and by comparing the achieved results with the current state of knowledge and potential applications. The findings contribute to a deeper understanding of how biomimetic principles can be effectively applied in the design and manufacturing of functional polymer surfaces for industrial practice using the technological approach presented here.

## 2. Materials and Methods

This section describes the creation of reliefs on molded parts using micro-machining technology, including the input material used, cutting conditions, and the machine and measuring equipment used to evaluate the created micro-reliefs. It also describes the process of preparing test samples using injection molding technology, including the polymers selected for production. Finally, the measuring equipment for determining wettability is presented.

### 2.1. Selection of Microstructures

Various types of microstructures imitating natural structures were used for experimental measurements. The proposed microstructures were selected according to the different complexity of the final geometry, both to verify the ability of micro-milling with a planned profile depth of 200 µm to produce these reliefs. It was also to verify the possibility of replicating the designed and prepared topologies for different types of polymer materials. The designed microstructures were first modeled in a CAD system, in Autodesk Inventor Professional 2024 (San Francisco, CA, USA) and Autodesk Fusion 360 2024 (San Francisco, CA, USA) software, and then produced using micro-milling technology. A total of three types of reliefs were designed and created.

**Figure 1:**
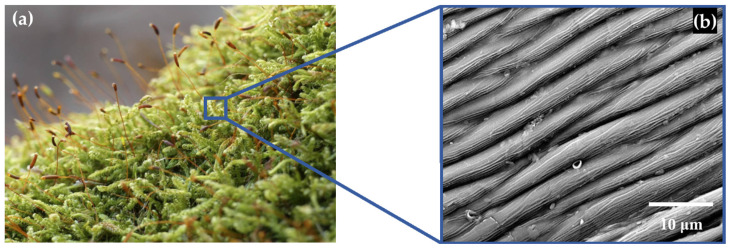
Relief No. 1—Rock Moss (*Hypnum cupressiforme*) [26] (**a**), SEM image of its surface [27] (**b**).

The first type of relief, designated as Relief No. 1, was wave-shaped and inspired by the surface of moss, specifically the surface of cypress-like moss (*Hypnum cupressiforme*). This type of surface in nature exhibits significant hydrophilic properties (see Figure 1).

**Figure 2:**
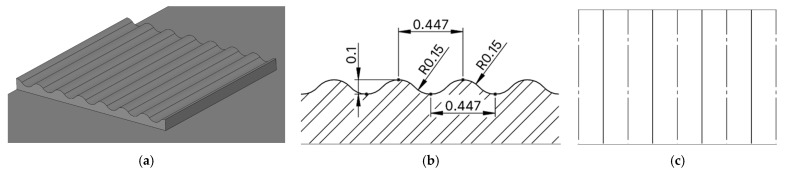
3D CAD image of the geometry of the proposed microstructure inspired by Rock Moss (*Hypnum cupressiforme*) (**a**), dimensions of the microstructure in cross-section (**b**), dimensions of the microstructure from above (**c**).

The dimensions of the selected profile were as follows: relief depth 0.1 mm, spacing between individual waves 0.447 mm, and arc radius in 0.15 mm (see Figure 2).

**Figure 3:**
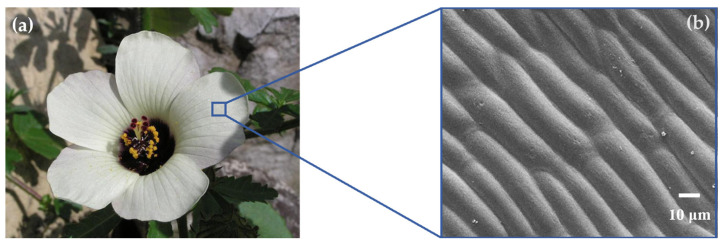
Relief No. 2—Three-Part Hibiscus (*Hibiscus trionum*) [28] (**a**), SEM image of its surface [29] (**b**).

The second type of relief, designated as Relief No. 2, was cone-shaped and inspired by the surface of the three-part hibiscus (*Hibiscus trionum*). Unlike moss, the surface structure on the petals repels water and thus exhibits significant hydrophobic properties (see Figure 3).

**Figure 4:**
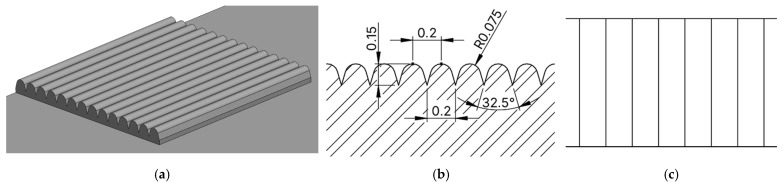
3D CAD image of the geometry of the proposed microstructure inspired by Three-Part Hibiscus (*Hibiscus trionum*) (**a**), dimensions of the microstructure in cross-section (**b**), dimensions of the microstructure from above (**c**).

The dimensions of the selected profile were as follows: profile depth 0.15 mm, spacing between individual elements 0.2 mm, and radius of individual elements 0.075 mm, see Figure 4.

**Figure 5:**
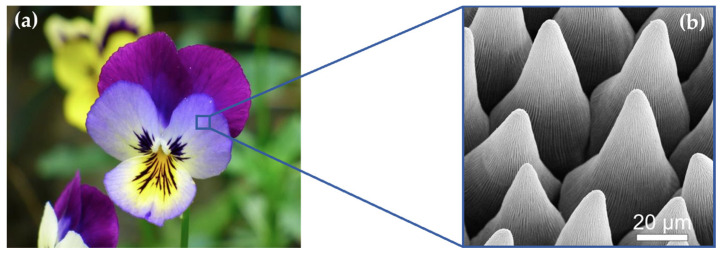
Relief No. 3—Heartsease (*Viola tricolor*) [30] (**a**), SEM image of its surface [31] (**b**).

The third type of relief, designated as Relief No. 3, was shaped like a truncated pyramid and was inspired by the surface of the flower of the three-colored pansy (*Viola tricolor*), see Figure 5.

**Figure 6:**
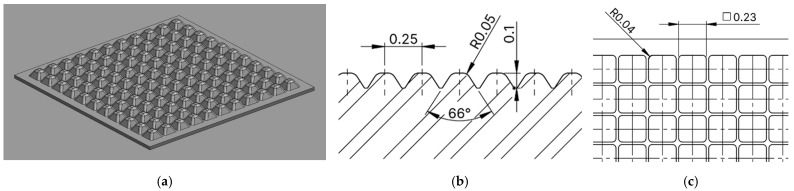
3D CAD image of the geometry of the proposed microstructure inspired by Relief No. 3—Heartsease (*Viola tricolor*) (**a**), dimensions of the microstructure in cross-section (**b**), dimensions of the microstructure from above (**c**).

The dimensions of the selected profile were as follows: profile depth 0.1 mm, spacing between individual elements 0.25 mm, lower base dimension 0.023 mm at an angle of 66°, see Figure 6.

### 2.2. Production of Microstructures by Micro-Milling

Micro-milling is a machining technique that can be used to create micro 3D shapes, reliefs, etc., with the required precision. This technology was selected because it is experimentally accessible through conventional CNC equipment, allows direct machining of metal base mold inserts, and enables rapid modification of surface topologies while maintaining high-dimensional accuracy and repeatability. However, micro-milling inherently limits the minimum achievable feature size to the order of tens of micrometers due to tool geometry. As a result, nanoscale hierarchical details typical of biological surfaces cannot be reproduced. These limitations can be overcome by alternative high-resolution texturing methods such as femtosecond-laser micromachining or lithographic nanoimprinting.

Due to the extremely high spindle speeds required for micro-milling (typically above 10,000 rpm), the cutting tools are subjected to significant wear during machining. Micro-milling was performed on a CMX 600 V three-axis vertical milling center from DMG MORI (Bielefeld, Germany), equipped with a SINUMERIK 840 D control system from Siemens (Munich, Germany). The machine spindle provides a maximum speed of 12,000 rpm. Because small-diameter tools require even higher rotational speeds for stable cutting, an external high-frequency electric spindle (CLASSIC 33 M, IMT GmbH, Gießen, Germany) was mounted directly onto the CNC machine via a clamping cone, allowing speeds of up to 80,000 rpm.

The material used for the production of microstructures on the molded parts of the mold was EN AW 7075 aluminum alloy supplied by AMAG (Ranshofen, Austria). This alloy was chosen for its excellent machinability (it produces small chips) and durability (the microstructures created by micro-milling are resistant to wear during replication). The basic mechanical properties of this alloy are as follows: yield strength R_p(0.2)_ 240 to 460 MPa, tensile strength R_m_ 360 to 540 MPa, elongation 2 to 8%, and hardness 104 to 160 HBW.

A double-edged spherical milling cutter made of sintered carbide with a diameter of 0.1 mm, a cutting edge radius of 0.05 mm, and a working length of 0.2 mm was selected for the production of microstructures. The helix angle was 30°, and the cutting part was coated with TiSiN. The cutting conditions for the creation of micro-reliefs were determined based on the recommendations of the micro-tool manufacturer, PM Tech (Prague, Czech Republic), with the machine spindle speed set at 60,000 rpm, the feed per tooth was 0.0007 mm, the feed rate was 80 mm/min, the cutting depth was 0.001 mm, and the cutting width was 0.005 mm.

### 2.3. Measuring Equipment

A VK-X1000 non-contact measuring device from Keyence (Osaka, Japan) was used to evaluate the accuracy and geometry of the micro-milled microstructures. This confocal microscope has a table with driven axes, allowing the reliefs to be observed and evaluated in terms of area and the surface to be scanned for subsequent evaluation. The measured data were analyzed using the MultiFileAnalyzer 2.1.3.89 software supplied by the manufacturer.

### 2.4. Polymer Materials for Structure Replication

Three materials were selected for the preparation of test samples in molds with designed and created reliefs. The selection was specifically chosen so that a non-hygroscopic, hydrophobic polymer, a hygroscopic, hydrophilic polymer, and a polymer with an amorphous or semi-crystalline structure were selected. Non-hygroscopic and semi-crystalline polypropylene (PP), hygroscopic and semi-crystalline polyamide (PA 6.6), and hygroscopic and amorphous acrylonitrile butadiene styrene (ABS) were selected.

Polypropylene (PP) was chosen for injection molding test samples primarily because of its non-polar nature. Specifically, polypropylene with the designation Mosten GB218 [32] manufactured by ORLEN Unipetrol RPA s.r.o. (Litvínov, Czech Republic) was chosen. Polyamide 6.6 (PA 6.6) is a semi-crystalline, highly polar thermoplastic that readily absorbs water both in direct contact and from the atmosphere. Polyamide 6.6 with the trade name Zytel ST801AHS BK010 [33] from DuPont de Nemours, Inc. (Wilmington, DE, USA) was selected. Acrylonitrile butadiene styrene (ABS) is an amorphous, moderately polar thermopolymer that was selected based on its polarity and amorphous structure. ABS Terluran GP-35 [34] manufactured by INEOS Styrolution Group GmbH (Frankfurt am Main, Germany) was selected. The main properties of selected polymers are in Table 1.

Due to the hygroscopicity of polyamide 6.6 (PA 6.6) and acrylonitrile butadiene styrene (ABS) polymers, these two materials were dried in a hot air dryer (SSD-40U/40H-CE, SHINI Plastic Technologies, Inc., Taipei, Taiwan) for 4 h at a temperature of 80 °C. In the case of polypropylene (PP), drying was carried out for 1 h at 60 °C to remove any moisture on the surface of the material.

### 2.5. Preparation of Test Polymer Bodies with Microstructures

The test samples were produced using injection molding technology on an Allrounder 320C Golden Edition injection molding machine from Arburg GmbH + Co KG (Lossburg, Germany). A two-plate quadruple injection mold with a cold runner system featuring a runner trap in the distribution channel, a film gate inlet into the mold cavities, and a central ejector using an ejector pin was used to inject the test samples. The molded specimens had dimensions of 40 × 40 × 3 mm. The size of the topological structure on the surface of the mold cavity was 6 × 6 mm. The thickness of the film gate was 1.4 mm, and the length of each branch of the distribution channel was 60 mm. The sprue had a conical shape with a diameter of 9.4 mm at the bottom, 6.0 mm at the top, and a total length of 58.5 mm. The mold cavity was equipped with inserts with surfaces imitating natural reliefs (see Section 2.1). The basic and constant parameters of the injection technology were as follows: mold temperature 60 °C (stabilized before each cycle and continuously controlled by a closed-loop water-circulation system), clamping force 500 kN, injection speed 20 cm^3^/s, back pressure during dosing 40 bar, packing pressure time 10 s, and total cycle time 60 s. In addition to the constant parameters, two technological parameters that have the greatest influence on the replication of the surface from the mold cavity to the surface of the plastic test sample were also changed: melt temperature and packing pressure. The melt temperature was changed in three levels with an increase of 20 °C between levels. The packing pressure was also changed in three levels with an increase of 150 bar between levels (see Table 2).

Before taking test samples by injection molding for the actual wettability measurements, several sets of test samples were injected for each combination of technological parameters used in the experimental measurements. These samples were discarded to ensure that the technological parameters stabilized at the required and set values. Five samples were then produced for each combination of technological parameters, selected material, and proposed relief.

### 2.6. Determination of Wettability

In this study, only static contact angles (CA) were measured, as the main objective was to evaluate the influence of polymer type, surface geometry, and processing parameters on overall wettability trends. This approach provides reliable comparative data; however, it does not allow for a full characterization of the wetting regimes or hysteresis behavior. Dynamic and roll-off angle measurements will therefore be included in future research to better distinguish between Cassie–Baxter and Wenzel states and to provide a more comprehensive analysis of surface functionality.

**Figure 7:**
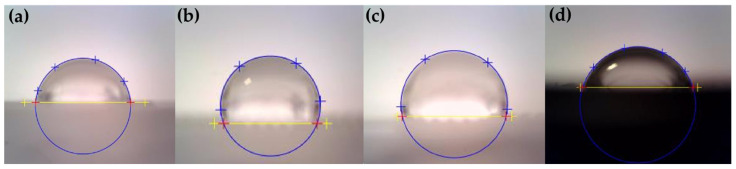
Example of an image of a droplet after its evaluation. Sample PP Relief No. 1, parameters (f) and measured contact angle 85.5° (**a**). Sample PP Relief No. 3, parameters (g) and measured contact angle 110.8° (**b**). Sample ABS Relief No. 3, parameters (h) and measured contact angle 103° (**c**). Sample PA 6.6 Relief No. 1, parameters (c) and measured contact angle 72.4° (**d**). The yellow line and yellow “+” symbols indicate the plane of the sample surface, the blue “+” symbols mark the droplet contour, and the red “+” symbols show the contact points between the droplet and the surface.

The wettability of the surfaces was measured at an air temperature of 23 ± 2 °C on the test specimens with replicas of natural structures. The measurement was performed using a See System E contact angle measuring device from Advex Instruments s.r.o. (Brno, Czech Republic). When measuring the contact angle of the liquid with the given relief, a drop of distilled water with a volume of 2 ± 0.06 μL was carefully applied to the surface using a Pipet4u Pro microvolume pipette from AHN Biotechnologie GmbH (Nordhausen, Germany). The device was then focused on this drop, and its image was saved in the manufacturer’s software, where the contact angle was subsequently evaluated by determining the surface plane on which the drop is in contact and the shape of the drop (see Figure 7). Contact angle measurements were performed on five test samples for the selected technological parameters, polymer materials, and designed and replicated reliefs.

## 3. Results

The topography and geometry of the surface at the micro scale are among the basic parameters that determine the degree of surface wettability by a liquid. For this reason, experimental measurements of wettability were performed on the designed and prepared relief geometries, which are replicated on the surface of plastic test specimens using injection technology. The experiment aimed to evaluate their influence on the hydrophobicity or hydrophilicity of the surface when using different types of polymer materials and set technological parameters. These properties were evaluated based on the measured average contact angles (see Table 3).

Experimental measurement is highly desirable, especially in cases where the influence and effect of the geometry of a replicated natural structure on the opposing wetting properties at the interface between the relief and the surface of the plastic test specimen is being evaluated. A typical example is the effect of relief, which is supposed to have hydrophilic properties (e.g., Relief No. 1—rock cypress), on the wettability of the surface of a plastic part made of a hydrophobic polymer (e.g., polypropylene). In other words, whether the surface remains hydrophobic and the relief, with its structure and geometry, has no impact on the change in wettability. Or, conversely, there will be a change to a hydrophilic surface, and the relief effect will be significant and desirable. The wettability properties of the manufactured replicas are therefore a very important factor in their evaluation and, at the same time, in deciding whether the replicas and their geometry, imitating natural surface structures, can be applied in technical practice at all. In order to compare the influence and effect of relief geometry on the wettability of plastic surfaces, the contact angle on a smooth flat surface was also measured for all measured samples (see Table 4).

**Figure 8:**
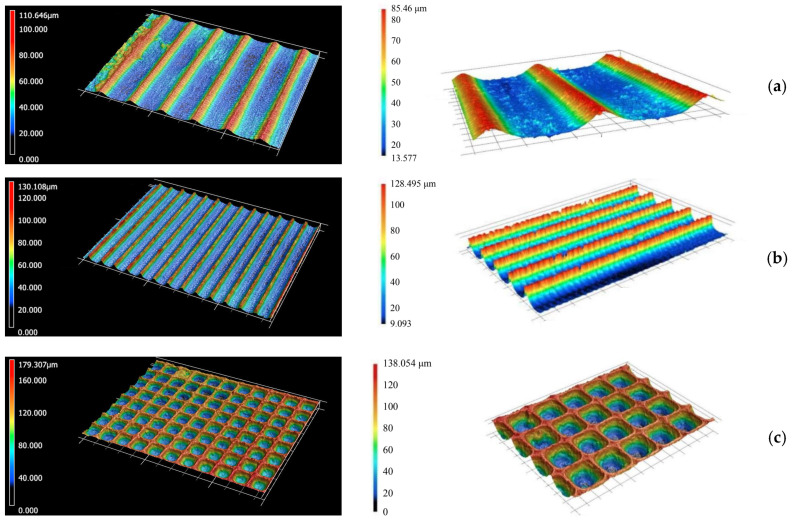
Three-dimensional images of the geometry and topology of microstructures on mold inserts (magnification 5×—left, 10×—right), Cypress-leaved moss (**a**), Three-part hibiscus (**b**), Three-color pansy (**c**).

Before measuring the wettability of the plastic bodies, the geometry and structure of the milled microstructures on the mold inserts were checked using a confocal microscope (see Section 2.3) to eliminate any damage or destruction of the relief. Images from confocal microscopy of the microstructures created on the surface of the mold cavity are shown in Figure 8.

**Figure 9:**
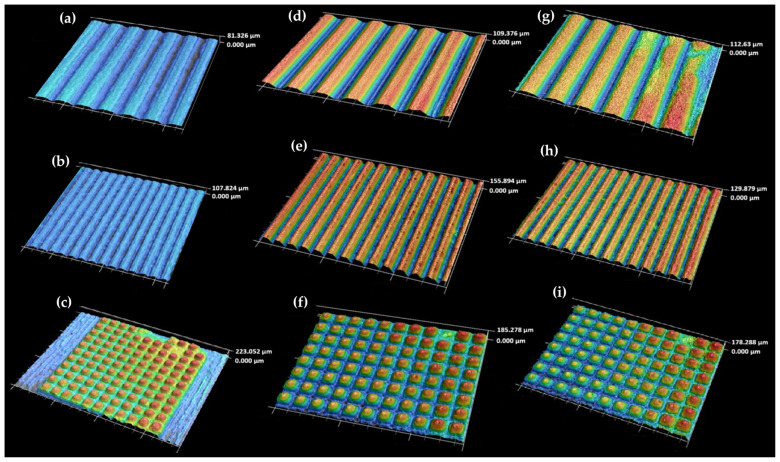
Three-dimensional images of microstructures reliefs on test samples (combination of parameters (i)). The combinations of polymer types and surface reliefs are as follows: PP and Relief No. 1 (**a**), PP and Relief No. 2 (**b**), PP and Relief No. 3 (**c**), ABS and Relief No. 1 (**d**), ABS and Relief No. 2 (**e**), ABS and Relief No. 3 (**f**), PA 6.6. and Relief No. 1 (**g**), PA 6.6. and Relief No. 2 (**h**), PA 6.6 and Relief No. 3 (**i**). The color mapping indicates the surface height distribution; warmer colors (red–yellow) represent elevated regions, while cooler colors (green–blue) correspond to lower surface areas.

In a similar manner, the surfaces of injection-molded plastic bodies for selected polymers and proposed relief geometries were also imaged using confocal microscopy prior to the actual contact angle measurement (see Figure 9).

## 4. Discussion

Contact angle values for relief no. 1 (Rock Moss), which is intended to imitate a structure with hydrophilic properties, were below 90° for almost all types of polymer materials and combinations of process parameters. Based on the contact angle values measured on the surface of the test plastic bodies imitating Rock Moss, it can therefore be concluded that this surface was hydrophilic for all polymers used to prepare the test samples. This result was also achieved in the case of the originally hydrophobic polypropylene (PP), where the application of the topology and structure of the Rock Moss surface to the surface of the sample changed the wettability of the surface. This phenomenon is also confirmed by a study by L. Lenshin et al. [39], who proposed several types of surface microstructures made by 3D printing using FFF technology from polylactic acid (PLA). Thanks to the hydrophilic microstructure, they achieved an increase in surface wettability on the samples depending on the geometry of the structure. It can therefore be concluded that the structure used and its relief geometry fulfilled their purpose and application goal. If we focus on the influence of temperature and pressure technological parameters for individual polymer materials on the contact angle value, in the case of polypropylene (PP), the size of the packing pressure has the greatest influence on the formation of a hydrophilic surface. For example, at a melt temperature of 220 °C, an increase in packing pressure (200 bar, 350 bar, 500 bar) leads to an increase in replication quality and thus a decrease in the contact angle. This phenomenon correlates with the research of Spenranz et al. [40], who studied the replication of nanostructures on polypropylene parts and concluded that the packing pressure has a significant effect on the final quality of the topological surface replication. In contrast, in test samples made of acrylonitrile butadiene styrene (ABS) with a hydrophilic structure, the contact angle increases with increasing melt temperature. This may cause polymer crystallization during sample production, resulting in relief deformation. In addition, this may also be due to its chemical composition and copolymer arrangement. To increase the hydrophilic properties of ABS, it is advisable to modify it, for example, with polyethylene terephthalate glycol (PETG), which significantly increases hydrophilic behavior, as reported in a study by Chen et al. [16]. Test samples made of polyamide 6.6 (PA 6.6) showed a decreasing contact angle at all three melt temperatures (290 °C, 310 °C, 330 °C) with increasing packing pressure (350 bar, 500 bar, 600 bar). The most significant downward trend was measured in contact angles at melt temperatures of 290 °C and 310 °C. At these temperatures and a packing pressure of 500 bar, the wetting angle of the sample with a hydrophilic relief decreased by up to 12% compared to the sample with a flat surface. The results therefore show that even in polyamide 6.6 (PA 6.6), the topological structure of Rock Moss had a positive effect, increasing the hydrophilicity of the surface.

The contact angle values for Relief No. 2, which was inspired by the hydrophobic surface of the three-part hibiscus, differ for the individual polymer materials used in the research. In test samples made of hydrophobic polypropylene (PP), the use of a hydrophobic relief mainly led to an increase in contact angles and thus to an enhancement of hydrophobic properties. The contact angle at melt temperatures of 200 °C and 220 °C increased with increasing packing pressure (200 bar, 350 bar, 500 bar). At a melt temperature of 240 °C, the contact angle did not change within the deviation range depending on the packing pressure. The situation was similar for amorphous acrylonitrile butadiene styrene (ABS), where the contact angle increased with packing pressure only at melt temperatures of 220 °C and 240 °C. For samples made of semi-crystalline polyamide (PA 6.6) at melt temperatures of 290 °C and 310 °C, a contact angle of less than 90° was measured. At lower temperatures, there was almost no change in the contact angle compared to a smooth surface. Meister et al. [41] state that, in addition to the mold temperature, the temperature of the plastic melt also has a significant influence on semi-crystalline polymers. The effect of the hydrophobic surface used was only apparent at a melt temperature of 330 °C for all packing pressures (350 bar, 500 bar, 650 bar), when a contact angle greater than 90° was measured. In the case of amorphous acrylonitrile butadiene styrene (ABS), the size of the packing pressure has a greater effect on the quality of replication, while in the case of semi-crystalline polyamide (PA 6.6), the effect of the melt temperature prevails.

The contact angle values for Relief No. 3, which was inspired by the hydrophobic surface of the tricolor pansy, were higher than 90°, and thus the surfaces of all test samples for all polymers and combinations of process parameters used achieved hydrophobic properties. The positive effect of a similar surface structure was also found in a study by Choo et al. [42], who replicated a rose-petal using UV-nanolithographic printing and also achieved hydrophobic properties on polyurethane acrylate (PUA) samples. If we were to evaluate the influence of temperature and pressure technological parameters, and their combinations, on the size of contact angles for individual polymer materials, then for polypropylene (PP), the increasing contact angle value is most influenced by the increasing packing pressure. When the packing pressure was increased from 200 bar to 500 bar (melt temperature 200 °C), the contact angle increased from 99.35 ± 3.36° to 108.35 ± 1.65°. The influence of packing pressure was also evident at medium melt temperatures (310 °C) in test samples made of acrylonitrile butadiene styrene (ABS). It therefore appears that in this case, the main parameter influencing replication was the size of the packing pressure. This is also confirmed by a study by Gamonal-Repiso et al. [10], who measured the replication of various structures produced by injection molding technology depending on technological parameters. At higher melt temperatures, the influence of packing pressure size is no longer entirely demonstrable. In the case of acrylonitrile butadiene styrene (ABS), both increasing the melt temperature and increasing packing pressure have a comparable effect on increasing the contact angle value. The conclusion is similar for polyamide (PA 6.6).

## 5. Conclusions

Surfaces with micro- and submicrostructures enable control of interfacial properties such as wettability, adhesion, and friction. This study examined the replication of biomimetic textures on polymer surfaces by injection molding using micro-milled aluminium alloy inserts, focusing on how polymer type, surface geometry, and processing parameters affect replication quality and wettability.

The results confirmed that the technological parameters of the injection molding process have a decisive effect on the quality of geometric replication and, consequently, on the resulting surface properties. Among these parameters, packing pressure and melt temperature were identified as the most influential factors. Higher packing pressures and moderate melt temperatures improved replication fidelity, leading to lower contact angles, particularly in semi-crystalline materials such as PA 6.6. Conversely, in ABS samples, the influence of melt temperature was more pronounced. The results showed that despite minor variations between individual materials, the general trends observed in this study are consistent with previously published findings, confirming that temperature and pressure play a dominant role in surface transfer during polymer processing.

Significant differences were also observed between individual polymers and surface geometries. The hydrophilic relief inspired by Rock Moss produced contact angles below 90° for all materials, including polypropylene (PP), which is naturally hydrophobic. This demonstrates that surface topology alone can substantially modify the wetting behavior of the polymer. In contrast, structures inspired by Hibiscus and Tricolor Pansy exhibited clearly hydrophobic properties, with contact angles exceeding 100–110°, depending on the processing parameters. These findings confirm that the selected surface geometries successfully replicated the functional characteristics of their natural counterparts.

From a technological standpoint, the research verified that micro-milling represents a practical and cost-effective method for manufacturing biomimetic surfaces on steel mold inserts. The technique offers high-dimensional accuracy, repeatability, and flexibility in surface design. However, its limitations in achieving nanoscale hierarchical features were acknowledged, and future work will focus on integrating high-resolution texturing techniques such as femtosecond-laser micromachining or lithographic nanoimprinting to achieve closer fidelity to natural multiscale surfaces. The presented findings contribute to a deeper understanding of how biomimetic principles can be effectively applied to the design and mass production of functional polymer components with tailored wetting properties.

## Figures and Tables

**Table 1:** Rheological and thermal properties of selected polymer materials [32,33,34].

	PP Mosten^®^ GB218 [32]	ABS Terluran^®^ GP-35 [34]	PA 6.6 Zytel^®^ ST801AHS BK010 [33]
Melt volume/flow rate	18 g/10 min (ISO 1133-1 [35])	34 g/10 min (ISO 1133-1 [35])	120 cm^3^/g (ISO 307 [36])
Molding shrinkage, parallel	1.76% (ISO 294-4 [37])	-	1.8% (ISO 294-4 [37])
Molding shrinkage, normal	1.93% (ISO 294-4 [37])	-	1.4% (ISO 294-4 [37])
Melting temperature (processing)	200–280 °C	220–260 °C	280–310 °C
Melting temperature (DSC)	168–172 °C (ISO 11357 [38])	-	262 °C (ISO 11357 [38])
Glass transition temperature	−10 °C (ISO 11357 [38])	105 °C (ISO 11357 [38])	75 °C (ISO 11357 [38])

**Table 2:** Values of the set parameters of melt temperature and back pressure for selected polymer materials.

		(a)	(b)	(c)	(d)	(e)	(f)	(g)	(h)	(i)
Melt temperature (°C)	PP	200	200	200	220	220	220	240	240	240
	ABS	220	220	220	240	240	240	260	260	260
	PA 6.6	290	290	290	310	310	310	330	330	330
Packing pressure (bar)	PP	200	350	500	200	350	500	200	350	500
	ABS	350	500	650	350	500	650	350	500	650
	PA 6.6	350	500	650	350	500	650	350	500	650

**Table 3:** Average values of measured contact angles (CA) for individual types of reliefs for selected polymer materials and injection conditions.

		(a)	(b)	(c)	(d)	(e)	(f)	(g)	(h)	(i)
PP	Relief No. 1	87.05 ± 1.76	88.23 ± 2.12	88.93 ± 1.63	88.27 ± 1.59	86.13 ± 1.61	84.55 ± 2.07	85.83 ± 2.24	84.80 ± 1.84	87.25 ± 2.36
	Relief No. 2	91.73 ± 2.27	91.55 ± 2.28	95.07 ± 1.85	90.43 ± 2.16	93.03 ± 1.64	00.97 ± 2.43	98.30 ± 1.53	96.33 ± 3.12	96.37 ± 1.74
	Relief No. 3	99.35 ± 3.36	106.20 ± 2.33	108.35 ± 1.65	101.85 ± 2.98	105.75 ± 2.19	108.02 ± 1.57	112.25 ± 2.67	109.73 ± 2.56	109.47 ± 2.25
ABS	Relief No. 1	78.03 ± 2.42	83.08 ± 3.65	74.80 ± 2.84	78.13 ± 2.37	81.60 ± 1.47	86.85 ± 2.09	85.23 ± 2.89	86.23 ± 2.51	81.93 ± 2.18
	Relief No. 2	82.65 ± 2.13	85.90 ± 1.98	89.18 ± 1.54	86.28 ± 1.37	92.20 ± 2.29	89.98 ± 3.17	94.28 ± 2.54	94.55 ± 2.78	88.48 ± 2.91
	Relief No. 3	94.73 ± 2.52	94.10 ± 2.23	96.95 ± 2.55	95.15 ± 1.76	96.65 ± 2.94	102.80 ± 3.41	98.13 ± 2.19	103.44 ± 3.47	101.27 ± 2.13
PA 6.6	Relief No. 1	82.35 ± 2.37	76.68 ± 2.26	72.28 ± 2.40	82.47 ± 2.09	78.70 ± 1.52	74.78 ± 3.17	90.38 ± 3.23	85.38 ± 1.99	83.98 ± 2.16
	Relief No. 2	82.45 ± 3.68	79.08 ± 3.18	79.60 ± 2.41	86.13 ± 2.79	87.35 ± 2.54	88.68 ± 2.40	94.48 ± 3.08	96.10 ± 1.91	96.55 ± 2.38
	Relief No. 3	98.43 ± 2.63	93.80 ± 3.84	98.75 ± 2.96	104.60 ± 3.14	99.58 ± 2.94	96.73 ± 2.58	98.90 ± 1.97	99.03 ± 2.53	97.40 ± 2.77

**Table 4:** Average values of measured contact angles (CA) on a smooth surface for selected polymer materials.

Polymer	PP	ABS	PA 6.6
Contant angle CA (°)	94.3 ± 1.98	83.5 ± 2.61	82.7 ± 2.24

## Data Availability

The original contributions presented in the study are included in the article, further inquiries can be directed to the corresponding author.
